# Pregnancies in young women with diagnosis and treatment of HER2-positive breast cancer

**DOI:** 10.18632/oncotarget.26611

**Published:** 2019-01-25

**Authors:** Matteo Lambertini, Giulia Viglietti

**Affiliations:** Department of Medical Oncology, U.O.C. Clinica di Oncologia Medica, Ospedale Policlinico San Martino, Genova, Italy; Department of Internal Medicine and Medical Specialties, School of Medicine, University of Genova, Genova, Italy

**Keywords:** breast cancer, pregnancy, HER2

Young breast cancer patients who have not yet completed their family planning at the time of diagnosis may still have pregnancy desire at the completion of adequate anticancer treatments and period of follow-up. Indeed, young women advocates have recently highlighted that pregnancy-related concerns have high priority [1]. Despite many physicians remain concerned that pregnancy in breast cancer survivors may have a detrimental prognostic effect by means of endocrine stimulation [2], increasing evidence has become available over the years on the safety of conceiving for patients with prior history of breast cancer diagnosis and treatment [3, 4]. However, there was lack of data on this regard to properly counsel young women with newly diagnosed HER2-positive breast cancer. In addition, considering both the suboptimal use of contraceptive methods among breast cancer patients [5] as well as the apparent lack of gonadotoxicity with the administration of trastuzumab (and/or lapatinib) [6], some physicians may need to counsel young women with HER2-positive breast cancer that have become accidentally pregnant during anti-HER2 targeted therapy. Considering that almost half of breast cancer physicians that have recently responded to a recent survey believe that first-trimester expsoure to trastuzumab is associated with high risk of malformations [2], many women in this situation are likely to be counseled about the need to terminate their pregnancy.

To investigate the prognostic effect of conceiving after diagnosis of HER2-positive early breast cancer and in order to provide evidence on the effect of exposure to the anti-HER2 agents trastuzumab and/or lapatinib on pregnancy outcomes, a recent analysis of all the pregnancy events that occurred as part of the NeoAdjuvant Lapatinib and/or Trastuzumab Treatment Optimization (NeoALTTO; NCT00553358) and the Adjuvant Lapatinib and/or Trastuzumab Treatment Optimization (ALTTO; NCT00490139) trials has been conducted [7]. Following study inclusion, among patients with ≤40 years at the time of diagnosis, 7 (7.5%) in the NeoALTTO trial and 85 (6.1%) in the ALTTO trial had a subsequent pregnancy, respectively. To assess the prognostic effect of conceiving after prior HER2-positive early breast cancer, the extended Cox model with time-varying covariates was used to account for guarantee-time bias by comparing disease-free survival between young patients with or without a subsequent pregnancy (this analysis included only women enrolled in the ALTTO trial). No significant difference in disease-free survival was observed between patients with or without a subsequent pregnancy (adjusted hazard ratio, 1.12; 95% confidence intervals, 0.52-2.42). Importantly, this analysis provides for the first time evidence on the safety of pregnancy in breast cancer survivors with HER2-positive disease and using data from a prospective randomized trial. In terms of pregnancy outcomes, out of 92 patients with at least one pregnancy after randomization in the two studies, 12 were unintentionally exposed to anti-HER2 therapy during gestation or shortly after its termination (exposed group) and 80 conceived following the completion of anti-HER2 targeted therapy (i.e. unexposed group). Among the 12 patients in the exposed group, 7 opted for an induced abortion and 5 decided to stop the treatment for continuing their pregnancy: all these 5 pregnancies resulted in live births with no congenital anomalies or pregnancy/delivery complications. Among the 80 patients in the unexposed group, 10 (12.5%) opted for an induced abortion and 10 (12.5%) had a spontaneous abortion; among the 54 (67.5%) patients who successfully completed their pregnancy, there were no pregnancy/delivery complications with the exception of one fetus (1.9%) with trisomy 21 (Down syndrome) [7].

There are two main messages deriving from this analysis. First, these findings reinforce the current recommendation that having a pregnancy after prior history of breast cancer should not be contraindicated after adequate treatment and follow-up [8, 9]. This statement appears to be valid in breast cancer survivors irrespectively of their tumor biology, including among women with HER2-positive disease. Second, although targeted therapies should not be actively administered during pregnancy as per current guidelines [8, 10], the limited data available with short accidental exposure to these agents during the first-trimester [11, 12] (including the recent findings from the NeoALTTO and ALTTO trials) may be partially reassuring about the low risk of congenital malformation. Therefore, pregnancy termination is probably not justified in this setting [13].

Results from the ongoing MotHER study (NCT00833963) collecting prospective information on patients exposed to anti-HER2 therapies during pregnancy as well as similar analyses from the large adjuvant trials that led to the approval of pertuzumab and neratinib in the early setting are awaited to provide further evidence in order to improve the counseling of young women with newly diagnosed HER2-positive breast cancer facing pregnancy-related concerns.
